# Learning What to See in a Changing World

**DOI:** 10.3389/fnhum.2016.00263

**Published:** 2016-05-31

**Authors:** Katharina Schmack, Veith Weilnhammer, Jakob Heinzle, Klaas E. Stephan, Philipp Sterzer

**Affiliations:** ^1^Department of Psychiatry and Psychotherapy, Charité - Universitätsmedizin BerlinBerlin, Germany; ^2^Translational Neuromodelling Unit, Institute for Biomedical Engineering, University of Zurich and ETH ZurichZurich, Switzerland; ^3^Bernstein Center for Computational Neuroscience, Charité - Universitätsmedizin BerlinBerlin, Germany; ^4^Berlin School of Mind and Brain, Humboldt-Universität zu BerlinBerlin, Germany

**Keywords:** visual perception, Bayesian brain, bistable perception, associative learning, sensory memory, hierarchical Gaussian filter

## Abstract

Visual perception is strongly shaped by expectations, but it is poorly understood how such perceptual expectations are learned in our dynamic sensory environment. Here, we applied a Bayesian framework to investigate whether perceptual expectations are continuously updated from different aspects of ongoing experience. In two experiments, human observers performed an associative learning task in which rapidly changing expectations about the appearance of ambiguous stimuli were induced. We found that perception of ambiguous stimuli was biased by both learned associations and previous perceptual outcomes. Computational modeling revealed that perception was best explained by a model that continuously updated priors from associative learning and perceptual history and combined these priors with the current sensory information in a probabilistic manner. Our findings suggest that the construction of visual perception is a highly dynamic process that incorporates rapidly changing expectations from different sources in a manner consistent with Bayesian learning and inference.

## Introduction

Sensory signals are inherently noisy and ambiguous. To make sense of such information, our perception strongly relies on expectations. For instance, when a photograph of a striped dress is consistent with two different color interpretations, the visual system resolves this ambiguity by using the expectation about how colors appear under common illumination conditions (Gegenfurtner et al., 2015; Lafer-Sousa et al., 2015). Such perceptual expectations about the appearance of a stimulus can be modified by repeated experience of the stimulus (Orbach et al., 1963; Maier et al., 2003; Chalk et al., 2010; Fischer and Whitney, 2014) or of associated cues (Sinha and Poggio, 1996; Ernst et al., 2000; Adams et al., 2004; Haijiang et al., 2006; Flanagan et al., 2008; Sterzer et al., 2008; Di Luca et al., 2010; Schmack et al., 2013). However, it has remained elusive how the visual system updates perceptual expectations moment by moment in a continuously changing world.

A powerful theory for the study of perceptual expectations is the Bayesian brain hypothesis (Knill and Pouget, 2004; Friston, 2005; Fiser et al., 2010; Seriès and Seitz, 2013). In this framework, perceptual expectations are formalized as prior probabilities that are combined with likelihoods representing sensory information by Bayes' rule. The resulting posterior probabilities reflect the outcome of perceptual inference about the causes of sensory inputs. In line with this idea, the influence of perceptual expectations on perception can be accurately explained by Bayesian inference (Weiss et al., 2002; Stocker and Simoncelli, 2006; Adams et al., 2010; Chalk et al., 2010; Acerbi et al., 2012; Zhang et al., 2013). Intriguingly, the outlined theoretical framework can also elegantly account for the update of perceptual expectations on the basis of ongoing perceptual experience: whenever a perceptual outcome deviates from a perceptual expectation, the resulting mismatch between the posterior and prior probability will trigger an update of the perceptual expectation. Thus, the Bayesian brain hypothesis affords a comprehensive mechanistic model not only for the influence of perceptual expectations on perceptual experience (inference) but also conversely for the influence of perceptual experience on perceptual expectations (learning).

Here, we applied the outlined Bayesian framework to investigate how perceptual inference is modulated by continuous learning from ongoing perceptual experience. To concurrently examine inference and learning, we devised a novel task using ambiguous and unambiguous visual motion stimuli in combination with audio-visual associative learning. Our task design allowed us to assess perceptual expectations based on perceptual history, i.e., previous perceptual outcomes, and associative learning, i.e., changing audio-visual contingencies. By the use of computational modeling, we quantified trial-by-trial learning of these perceptual expectations and the effect of these perceptual expectations in perceptual inference. We hypothesized to find evidence for a continuous integration of different sources of experience in the moment-to-moment updating of perceptual expectations, thus resulting in the flexible adaptation of perceptual inference to dynamic changes in the environment.

## Materials and methods

### Participants

Participants that were included into analyses were capable of stereopsis and of perceiving the ambiguous sphere stimulus in both possible configurations (see below). Experiment 1 was completed by 31 participants (age range 19–61 years, age median 25 years, 19 female). Experiment 2 was completed by 31 participants who had not participated in Experiment 1 (age range 20–33 years, median 24 years, 20 female). All participants of both experiments were naive as to the purpose of the study. All of them had normal or corrected-to-normal vision. All participants gave written informed consent in accordance with the Declaration of Helsinki beforehand, and the experiments were approved by the ethics committee of Charité - Universitätsmedizin Berlin.

### General method

**Figure 1:**
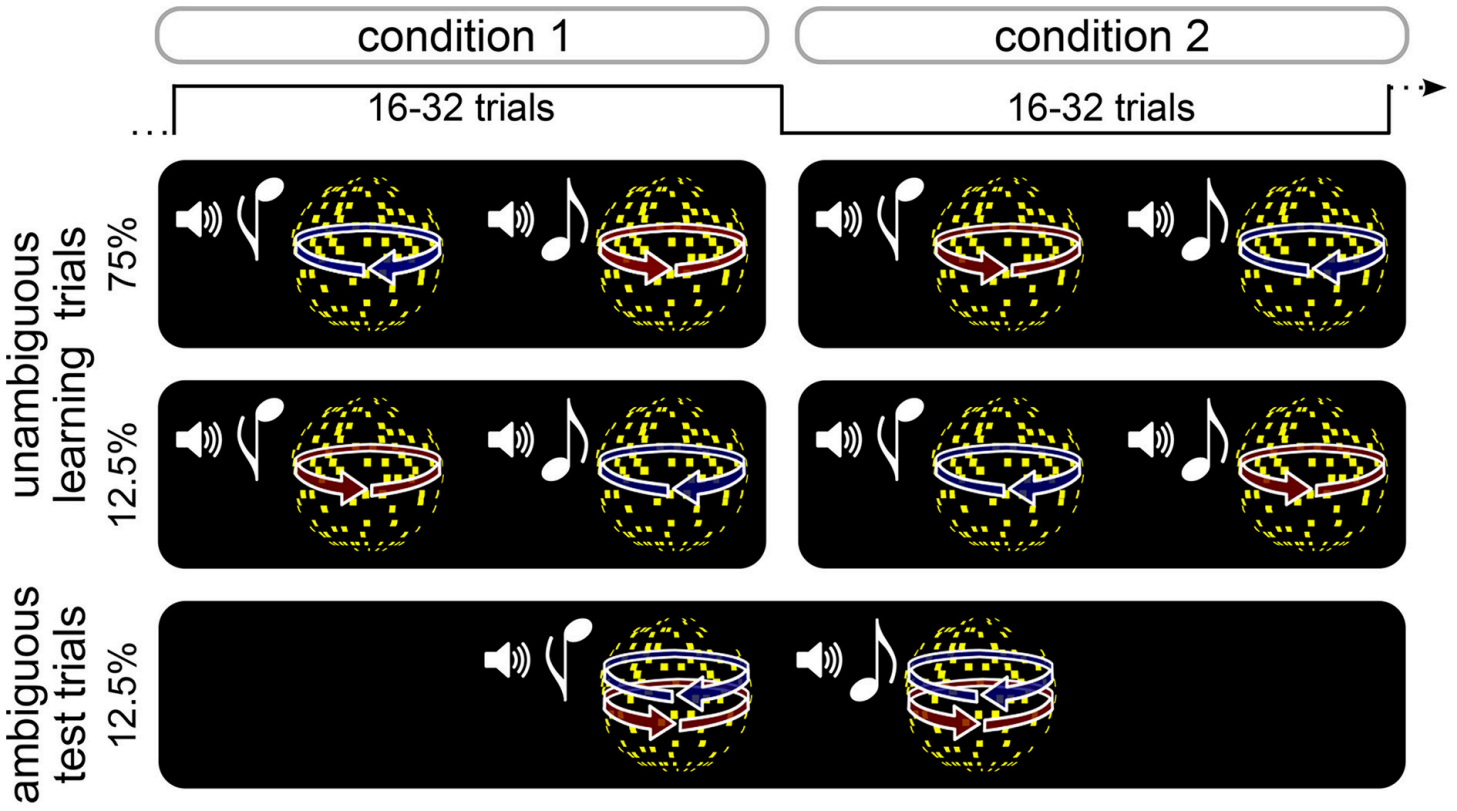
**Schematic illustration of experimental design**. Participants performed an probabilistic reversal learning task in which tones were coupled to the rotation direction of a sphere stimulus. On unambiguous learning trials, the direction of rotation was unambigiously determined by 3D disparity cues. The association between tones and rotation direction was probabilistic and changed unpredictably every 16–32 trials. On interspersed ambiguous test trials, the sphere stimulus was perceptually very similar to the one in the training trials but lacked 3D disparity cues and could therefore be perceived either in one or the other rotation direction. After each trial, participants indicated the perceived rotation direction as quickly and accurately as possible, and reported their confidence about the percept.

Participants performed an associative reversal learning task in which different kinds of rapidly changing expectations about the appearance of an ambiguous stimulus were induced (Figure 1, see below). High or low tones were associated with leftward or rightward rotation of a subsequently presented visual sphere stimulus. On learning trials, the direction of rotation was determined by disparity cues that yielded an unambiguous 3D appearance of the rotating sphere. The association of tones with rotation directions was probabilistic and changed unpredictably every 16–32 trials. Pseudo-randomly interspersed were test trials, on which the sphere lacked disparity cues and was thus ambiguous with respect to rotation direction, but indistinguishable in appearance from the unambiguous 3D stimulus. After each trial, participants indicated the perceived rotation direction as quickly and accurately as possible, and reported their confidence about the percept. Visual and auditory stimuli were produced using Matlab (MathWorks Inc.) and Cogent 2000 toolbox (http://www.vislab.ucl.ac.uk/cogent.php). Visual stimuli were presented on a on a CRT monitor (1024 × 768 pixels resolution, 60 Hz frame rate). In order to produce stereoscopic vision in the unambiguous training trials (see below), all stimuli were displayed dichoptically through a mirror stereoscope.

### Experiment 1

Trials started with the presentation of either a high-pitched or a low-pitched tone (500 or 1250 Hz, duration 0.3 s) and a black screen with a central white fixation cross. After a variable interval (0.1–0.3 s), a sphere appeared (diameter 10.6° of visual angle). The sphere rotated along a vertical axis at 1/15 Hz. It consisted of 450 randomly distributed yellow square “dots” (maximum size 0.2° × 0.2°) moving coherently left- or rightward on a black background with a central fixation cross. In training trials, the sphere contained interocular disparity cues (maximal offset 0.5°) that were induced with a mirror stereoscope, thereby rendering rotation direction unambiguous. In test trials, the sphere was shown without disparity cues and rotation direction was therefore ambiguous. For naive observers, the ambiguous and the unambiguous sphere are nearly indistinguishable in appearance. In all trials, the sphere was displayed until participants indicated the perceived rotation direction (left or right) by pressing a key on a computer keyboard (left arrow or right arrow, respectively). After the presentation of a blank screen for 0.4 s, participants reported their confidence about their decision (very sure, sure, unsure, very unsure). After an interval jittered between 0.35 and 1.65 s the next trial started. On every trial, we recorded the perceived rotation direction (left or right), the reaction time for this response, as well as the confidence rating (very sure, sure, unsure, very unsure).

Participants performed a total of 576 trials divided in six runs. Tones and rotation directions were associated probabilistically, and the association was reversed between trial blocks consisting of 16, 24, or 32 trials (see Figure 1). In half of the trial blocks, 75% of all trials were unambiguous training trials in which the high-pitch tone was followed by a left-ward rotating sphere (and a low-pitch tone by right-ward rotating sphere), and 12.5% of the trials were unambiguous training trials the high-pitch tone was followed by a right-ward rotating sphere (and a low-pitch tone by left-ward rotating sphere). In the other half of trial blocks, unambiguous training trials were presented with reversed associations. In all trial blocks, 12.5% of the trials were test trials during which both high-pitch and low-pitch tones were followed by a sphere stimulus with ambiguous rotation direction. Participants were told that the relation between tones and rotation direction was probabilistic and that this probabilistic relation would change unpredictably over time. They were not informed about the intervals over which the probabilistic relation changed or about the occurrence of ambiguous stimuli. Trial order was pseudo-randomized, ensuring that ambiguous test trials were preceded by at least three unambiguous training trials with the currently more probable tone-rotation association.

Two additional individuals that had performed Experiment 1 were not included into analyses because their performance in the discrimination of the unambiguous cues was at chance level indicating impaired stereopsis. Another additional individual that had performed Experiment 1 was not included into analyses due to always seeing the ambiguous sphere rotating in the same direction indicating that this individual might not have been capable of perceiving the ambiguous stimulus in both possible configurations.

### Experiment 2

In Experiment 1, the task was kept as easy as possible and a fixed mapping between the key presses and perceived rotation direction was used throughout the whole experiment, i.e., participants always indicated “right” with the right arrow key and “left” with the left arrow key. This raises the possibility that any observed effects of perceptual expectations on reported perception partly reflect a bias in key pressing behavior rather than a bias in perceptual inference. To rule out this possibility, we conducted a second experiment in which participants' key presses were uncorrelated with their perception. Please note that this experimental manipulation comes at the cost of increased task demands with possibly increased error rates and noisier response speeds.

The design of Experiment 2 closely resembled the design of Experiment 1 with the following exception. When the sphere appeared, participants judged the perceived rotation direction in mind and pressed the space bar on a keyboard as soon as they had made their decision. Then, two symbols were displayed next to each other, one pointing to the left and one pointing to the right. The task was to indicate whether the symbol displayed at the right or the symbol at the left matched the perceived rotation direction by pressing the right arrow key or the left arrow key, respectively. The position of the symbols was pseudo-randomized so that the left arrow key corresponded to left rotation direction in half of the trials and to right rotation direction in the other half of the trials. After participants had made their choice, the trial proceeded as in Experiment 1 with the confidence rating. On every trial, we recorded the reaction time for the space bar press indicating that the participant had made a decision, perceived rotation direction (left or right) as well as the confidence rating (very sure, sure, unsure, very unsure).

One additional individual that had performed Experiment 2 was not included into analyses because the individual's performance in the discrimination of the unambiguous cues was at chance level indicating impaired stereopsis. Two additional individuals that had performed Experiment 2 were not included into analyses due to always seeing the ambiguous sphere rotating in the same direction indicating that these individuals might not have been capable of perceiving the ambiguous stimulus in both possible configurations.

### Measurement of perceptual biases

The measurement of expectation-induced perceptual biases was based on the perceptual assessment of the ambiguous figures. To derive these perceptual biases, we defined distinct perceptual expectations from the sources associative learning and perceptual history for each ambiguous test trial. For associative learning, the current perceptual expectation was determined by the pitch of the tone and the currently predominant association, i.e., in phases of the experiment during which high-pitch tones was followed by a rightward rotation direction in 75% of the trials, we assumed that on a trial that started with a high-pitch tone rightward rotation direction was expected. Please note that on ambiguous test trials the currently predominant association was equivalent to the recently experienced association, as our task design ensured that each ambiguous test trial was preceded by at least three unambiguous training trials matching the currently predominant association (see Methods, Experiment 1). For perceptual history, we followed the previously formulated distinction between the perceptual effects of previous unambiguous visual stimulation (“priming”) and previous ambiguous visual stimulation (“sensory memory”; Pearson and Brascamp, 2008). For priming, the current perceptual expectation was set equal to the perceived rotation direction in the unambiguous trial that directly preceded the ambiguous trial, i.e., after an unambiguous trial with a rightward rotating sphere stimulus, we assumed that rightward rotation was expected. For sensory memory, the current perceptual expectation was defined by the perceived rotation direction in the last ambiguous trial, i.e., after an ambiguous trial in which rightward rotation was perceived, we assumed that on the next ambiguous trial rightward rotation was expected. Please note that the factor associative learning describes the coupling between tones and perceptual outcomes (the contingency level), whereas priming and sensory memory are defined by the perceptual outcomes alone (the perceptual level). As a consequence, the perceptual expectations induced by “associative learning” and by “priming” are orthogonal to each other. For any of the perceptual expectations (associative learning, priming, or sensory memory), the magnitude of the perceptual bias was then calculated as the difference between the percentage of ambiguous trials in which rotation direction was perceived in congruence with the current perceptual expectation and the percentage of ambiguous trials in which rotation direction was perceived in incongruence with the current perceptual expectation.

To further characterize the nature of the perceptual bias induced by associative learning, we calculated the number of trials that were rated at the highest of the four confidence levels (“very sure”), and repeated the analysis of the perceptual bias induced by associative learning for only these high-confidence trials. Moreover, we conducted a two-way ANOVA that tested for the effects of stimulus type (“ambiguous” vs. “unambiguous”) and associative learning (“congruent” vs. “incongruent”) on the number of high-confidence trials.

We further aimed to relate the perceptual bias induced by associative learning to the effect of associative learning on motor response speed. Specifically, we assumed that on unambiguous training trials participants would respond faster if they had predicted the upcoming perceived direction of rotation, and that such motor adaptation would constitute an indirect measure of associative learning. We hypothesized that participants with stronger motor adaptation in unambiguous training trials would also exhibit a stronger perceptual bias in ambiguous test trials. To quantify the motor adaptation induced by associative learning, we calculated the difference in mean reaction times between unambiguous trials that were congruent with the expectation induced by associative learning and that were incongruent with the expectation induced by associative learning. In order to correct for individual differences in absolute reaction times, this difference was divided by the mean reaction times in all unambiguous trials. Please note that as a result of this normalization procedure the motor adaptation measure does not have a unit. In both experiments reaction times were defined by the key press that indicated that participants had made their perceptual choice, i.e., in Experiment 1 by presses of the left arrow or right arrow key and in Experiment 2 by presses of the space bar.

To verify that participants' changing expectations induced by associative learning had an effect on perception independently from priming and sensory memory, we conducted a trial-wise repeated measures logistic regression. A generalized linear model was fitted to the binary dependent variable coding perceptual outcomes. The model included three regressors of interest coding the binary perceptual expectations from associative learning, priming, and sensory memory. The model specification assumed a Poisson distribution for the binary predictors, and a log link function relating perceptual outcomes to a linear combination of the predictors. Statistical significance of the betas of each of the regressors was assessed by the Wald-Chi-square test. Following the recommendation of one of our reviewers, we compared the full model including all three regressors with reduced models that lacked one or two of the regressors in order to further assess whether associative learning, priming and sensory memory had independent effects on perception. To this end, model fit was quantified by the quasi-likelihood under independence criterion (QIC) as implemented in SPSS.

For all statistical analyses we used the Statistics Toolbox of Matlab (MathWorks Inc.) with exception of the trial-wise repeated measures logistic regression that was conducted in SPSS (IBM SPSS Statistics, Version 23.0.0.0). Prior to analysis, incorrect or missed responses were excluded. Please note that by definition there were no incorrect responses on ambiguous test trials. For experiment 1, on average 1.6 ± 0.4 (mean ± s.e.m) trials were excluded per participant. For experiment 2, on average 0.8 ± 0.2 (mean ± s.e.m) trials were excluded per participant.

### Bayesian modeling

In our experiments, participants did not have direct access to the hidden contingency between tones and perceptual outcomes and had to infer the association over time. Using the currently predominant association (hidden contingency) in the logistic regression model thus constitutes a simplification. We therefore adopted a computational approach that specifically addresses this problem by explicitly modeling the individuals' belief trajectory about this hidden contingency in order to generate trial-by-trial perceptual expectations induced by associative learning. To enable the integration of perceptual expectations induced by associative learning with perceptual expectations based on perceptual history, we used a Bayesian framework that frames perception as an inferential processes in which perceptual decisions are based on posterior distributions. According to Bayes' rule, such posterior distributions are derived from likelihood distributions representing the sensory information, and prior distributions, which—in the context of this experiment—can be used to formalize expectations about perceptual outcomes. Crucially, such priors can stem from different sources: “priming” (the influence of a visual percept on the subsequent trial) and “sensory memory” (the influence of the visual percept in an ambiguous trial on the subsequent ambiguous trial) can be framed as priors for visual perception that are derived from perceptual history. Furthermore, such priors can be constructed by “associative learning,” i.e., the participants' continuously updated belief about the probabilistic coupling between tones and visual stimuli. All priors can be modeled by Gaussian probability distributions which are defined by their respective mean and precision (the inverse of variance). Importantly, the precision term represents the impact of a prior on the posterior distribution and thus relates to its influence on visual perception.

The modeling analysis presented here (see **Figure 3** for a schematic overview) was based on the prediction of perceptual outcomes, i.e., on the perception of left- or rightward rotation, from specific prior distributions and the likelihood. In brief, the prior distributions “associative learning,” “priming,” and “sensory memory” were parametrized by means (μ) and precisions (π). The prior distribution “associative learning” (μ_*a*_ and π_*a*_) was defined by an ideal Bayesian learner that is derived by a Hierarchical Gaussian Filter (HGF, Mathys et al., 2014, HGF 4.0 toolbox, www.translationalneuromodeling.org). The prior distributions “priming” (μ_*p*_ and π_*p*_) and “sensory memory” (π_*s*_ and μ_*s*_) were defined by the respective perceptual history. The prior distributions were computed into a joint prior distribution (μ_*prior*_ and π_*prior*_). In order to determine which prior distributions influence perceptual outcomes, different models including all possible combinations of the prior distributions “associative learning,” “priming,” and “sensory memory” were compared by Bayesian model selection.

On each trial, an ambiguous or unambiguous visual stimulus was presented which could be perceived as rotating either right- or leftwards. These two alternative visual percepts were given by:

(1) $$\begin{array}{r} \theta =\{\begin{array}{ll} 1: & \;\to \;(rotation)\\ 0: & \;\leftarrow \;(rotation) \end{array} \end{array}$$

To formalize the sensory information on both ambiguous and unambiguous trials, we constructed a likelihood by combining a a fixed bimodal distribution (Sundareswara and Schrater, 2008) with a distribution reflecting the stereodisparity between the monocular channels that disambiguated the visual spheres. The mean of the distribution “stereodisparity” in trial *t* was defined by stereodisparity between the two eyes in trial *t*:

(2) $$\begin{array}{l} \mu_{stereo}(t)\{\begin{array}{ll} 1: & \;\to \;(disambiguation)\\ 0.5: & \;↔\;(ambiguous)\\ 0: & \;\leftarrow \;(disambiguation) \end{array} \end{array}$$

In order to define the prior distribution “associative learning,” we defined by an ideal Bayesian learner by using HGF for binary inputs without perceptual uncertainty (Mathys et al., 2014), as implemented in the HGF 4.0 toolbox (distributed within the TAPAS toolbox, www.translationalneuromodeling.org). Importantly, we used the HGF on contingencies between tones and rotation directions and not on rotation directions *per se*. The HGF therefore represented a model of optimal higher-order learning of changing contingencies. In an initial step, we estimated the parameters of the HGF by inverting this generative model based on the stimulus sequence alone, under default assumptions of the HGF. In other words, we determined those parameter values under which the stimulus sequence was least surprising, given the belief trajectories of the HGF. This yielded an ideal Bayesian learner accounting for the associative learning effects in our paradigm; the ensuing trial-wise predictions were integrated with predictions from other sources (“priming,” “sensory memory”) into a Bayesian model of behavior described below.

The contingencies emerging from the probabilistic coupling of tones and rotations were defined as follows:

(3) $$\begin{array}{r} Inputs=\{\begin{array}{ll} 1 & \;for\;↑\;(tone)\;+\;\leftarrow \;(rotation)\\ 1 & \;for\;↓\;(tone)\;+\;\to \;(rotation)\\ 0 & \;for\;↑\;(tone)\;+\;\to \;(rotation)\\ 0 & \;for\;↓\;(tone)\;+\;\leftarrow \;(rotation) \end{array} \end{array}$$

The mean of the prior distribution “associative learning” on trial *t* was defined by:

(4) $$\begin{array}{l} \mu_{a}(t)=\{\begin{array}{ll} {\hat{\mu }}_{1}(t): & \;for\;↓\;(tone)\\ 1-{\hat{\mu }}_{1}(t): & \;for\;↑\;(tone) \end{array} \end{array}$$

The mean of the prior distribution “priming” on trial *t* was defined by the visual percept on the preceding trial:

(5) $$\begin{array}{l} \mu_{p}\left(t\right)=\theta \left(t-1\right) \end{array}$$

The mean of the prior distribution “sensory memory” on trial *t* was defined by the visual percept on the preceding ambiguous trial *t*_*a*_:

(6) $$\begin{array}{l} \mu_{s}\left(t\right)=\theta \left(t_{a}\right) \end{array}$$

In order to predict the perceptual outcomes, we derived the posterior distribution with respect to left- or rightward-rotation from the model. This distribution results from a “stereodisparity”-weighted bimodal likelihood distribution (Sundareswara and Schrater, 2008) by a combination of prior distributions such as “associative learning,” “priming,” and “sensory memory.”

For a specific combination of these prior distribution, a joint prior distribution with mean μ_*prior*_ and precision π_*prior*_ can be calculated by adding up the means of influencing factors relative to their respective precision:

(7) $$\begin{array}{l} \mu_{prior}=\frac{\pi_{a}\mu_{a}+\pi_{p}\mu_{p}+\pi_{s}\mu_{s}}{\pi_{prior}} \end{array}$$

(8) $$\begin{array}{l} \pi_{prior}=\pi_{a}+\pi_{p}+\pi_{s} \end{array}$$

To derive the posterior distribution, this joint prior distribution is combined with the likelihood. To this end, the joint prior distribution (parametrized by μ_*prior*_ and π_*prior*_) is used along with the the “stereodisparity” information of the bimodal likelihood distribution (parametrized by μ_*stereo*_ and π_*stereo*_) to adjust the probability ratio *r* of percept θ_1_ and θ_2_ in a bimodal distribution (Sundareswara and Schrater, 2008):

(9) $$\begin{array}{l} \mu_{m}=\frac{\pi_{prior}\mu_{prior}+\pi_{stereo}\mu_{stereo}}{\pi_{m}} \end{array}$$

(10) $$\begin{array}{l} \pi_{m}=\pi_{prior}+\pi_{stereo} \end{array}$$

(11) $$\begin{array}{l} r=\frac{P\left(\theta_{1}\right)}{P\left(\theta_{2}\right)}=exp\left(\frac{{\left(\theta_{1}-\mu_{m}\right)}^{2}-{\left(\theta_{2}-\mu_{m}\right)}^{2}}{\pi_{w}^{-2}}\right) \end{array}$$

(12) $$\begin{array}{l} P\left(\theta_{1}\right)=\frac{1}{r+1} \end{array}$$

Note that whether the prior distributions (parametrized by μ_*prior*_ and π_*prior*_) and the stereodisparity information of the likelihood (parametrized by μ_*stereo*_ and π_*stereo*_) are integrated at once or sequentially cannot be disentangled based on the model presented here, since both scenarios are mathematically equivalent, hence leading to identical posterior distributions and identical predictions of behavior.

The predicted response *y* of the subjects is given by applying a unit sigmoid function with ζ = 1 to *P*(θ_1_):

(13) $$\begin{array}{l} y=\frac{P{\left(\theta_{1}\right)}^{\zeta }}{P{\left(\theta_{1}\right)}^{\zeta }+{\left(1-P\left(\theta_{1}\right)\right)}^{\zeta }} \end{array}$$

In order to determine which prior distributions influenced perceptual outcomes, we first constructed models incorporating all combinations of the prior distributions “associative learning,” “priming,” and “sensory memory.” To this end, the precisions of these distributions were either estimated as free parameters in the perceptual model or fixed at zero (thereby effectively removing a prior distribution from the model). Furthermore, the precision of the likelihood-weighing distribution “stereodisparity” was always estimated as a free parameter. Thus, this yielded 2^3^ = 8 models. After identifying the optimal model using Bayesian model selection, we extracted its posterior parameters with regard to the respective precision of the prior distributions, and related them to the behavioral measures. Crucially, we calculated the Pearson's correlation coefficient for the relationship between π_*a*_ encoding the precision of the prior distribution “associative learning” and the behavioral perceptual bias induced by associative learning.

We employed a Variational Bayesian model inversion in order to estimate the free parameters of the model. This procedure determines the posterior distributions over parameters by maximizing the log-model evidences. This translates onto the negative surprise about an individual subject's data on the background of a specific model, and is approximated by negative free energy (Friston and Stephan, 2007). As an optimization algorithm, we chose the quasi-Newton Broyden-Fletcher-Goldfarb-Shanno minimization algorithm, as implemented in the HGF 4.0 toolbox. For model inversion, the prior distributions for the free parameters (not to be confounded with the prior distributions in the perceptual model) were modeled as log-normal distributions. The parameters π_*a*_, π_*p*_, and π_*s*_ were included into a model by setting the respective prior mean to log(0.5) and the prior variance to 1. To exclude any of the parameters π_*a*_, π_*p*_, and π_*s*_ from a model, the respective prior mean was fixed to log(0) by setting the prior variance to 0. The parameter π_*stereo*_ was always estimated with a prior mean of log(2.5) and a prior variance of 1. All other parameters were fixed and were set to: μ_2_ = 0, μ_3_ = 0, ρ_2_ = 0, κ_2_ = 1, ω_2_ = −2.28, ω_3_ = −6.14, ζ = 1.

Following the suggestion of one of our reviewers, we repeated the Bayesian modeling analyses with an alternative formulation of the prior distribution “associative learning,” and replaced the ideal Bayesian learner by Bayesian learning models that were fitted to the individuals' behavior. To this end, the parameters that control the time-invariant component of the learning rate at the second and third level were estimated as additional free parameters: ω_2_ (prior mean: -2.28, prior variance: 1) and ω_3_ (prior mean: -6.13, prior variance: 1). All other parameters were kept free or fixed as described above. This alternative formulation of the prior distribution “associative learning” allowed us to generate belief trajectories that take into consideration inter-individual differences in learning rate.

## Results

### Experiment 1: expectation-induced perceptual biases

**Figure 2:**
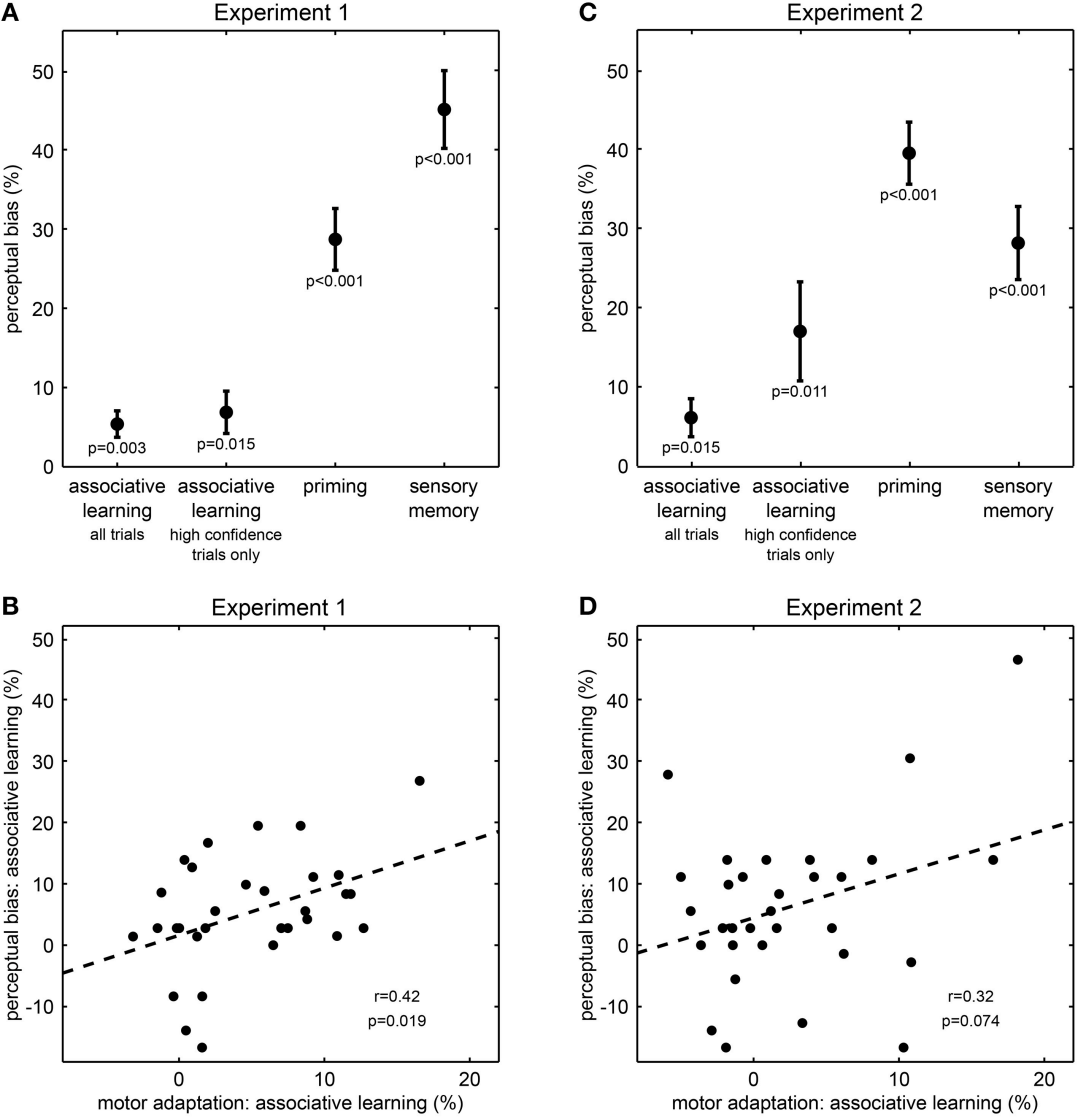
**Results from Experiment 1 and Experiment 2**. **(A,C)** Effect of perceptual expectations on visual perception (mean ± s.e.m.). Perceptual biases quantify the influence of perceptual expectations from associative learning, priming or memory on perception in ambiguous test trials. *P*-values result from one-sample *t*-tests. Please note that the influence of associative learning corresponds to effects of intermediate size (all Cohen's *d* > 0.5). **(B,D)** Correlation across participants between perceptual bias and motor adaptation for associative learning. Motor adaptation describes the effect of associative learning on reaction times in unambiguous training trials. Each dot represents one participant; the dashed line illustrates the fitted regression line; *r*- and *p*-values result from Pearson's product-moment correlations.

We first examined whether perceptual history, i.e., the previous experience of the visual sphere stimuli had an impact on perception. In line with previous work (Pearson and Brascamp, 2008), we found perception on the ambiguous test trials to be biased toward the rotation direction of both the preceding unambiguous training trial (“priming,” 28.7% ± 3.9 s.e.m., difference between percentages of trials perceived in same and opposite direction, *t*_(30)_ = 7.37, *p* < 0.001, one-sample *t*-test, Figure 2A) and the preceding ambiguous trial (“sensory memory,” 45.1% ± 4.9 s.e.m., *t*_(30)_ = 9.19, *p* < 0.001, Figure 2A). Thus, the qualitative perception of the ambiguous sphere stimuli strongly depended on previous perceptual outcomes, indicating that perceptual expectations substantially contribute to resolve ambiguity in visual information and are flexibly updated by perceptual history.

Next and most importantly, we tested whether the rapidly changing contingencies between tones and visual sphere stimuli affected perception. Notably, ambiguous test trials stimuli were perceived more frequently as rotating in the direction consistent with the currently predominant association than in the direction inconsistent with this association (“associative learning,” 5.4% ± 1.7 s.e.m., *t*_(30)_ = 3.23, *p* = 0.003, Figure 2A), and the size of this effect corresponded to an intermediate to strong effect (Cohen's *d* = 0.6). To investigate whether this effect of associative learning was due to a generally lower confidence in the perception of ambiguous stimuli, we analyzed the confidence rating data in a two-way ANOVA (Supplementary Figure S1). This analysis revealed a main effect of stimulus type on confidence ratings, indicating that on ambiguous test trials compared to unambiguous training trials participants indicated less frequently the highest confidence level (*F* = 11.9, *p* = 0.002). There were no main effect of associative learning (*F* = 1.6, *p* = 0.22) nor an interaction between stimulus type and associative learning (*F* = 0.4, *p* = 0.55). Despite the main effect of stimulus type, participants still rated more than 75% of the ambiguous test trials at the highest confidence level (Supplementary Figure S1), suggesting that the vast majority of ambiguous test trials was perceptually indistinguishable from unambiguous test trials. Most importantly, when considering only these high-confidence ambiguous test trials, we still found that stimuli were perceived more frequently as rotating in the direction consistent with the currently predominant association than in the direction inconsistent with this association (6.9% ± 2.7 s.e.m., *t*_(30)_ = 2.57, *p* = 0.015, Cohen's *d* = 0.5, Figure 2A). Thus, our results so far suggest that perception of the ambiguous sphere stimuli was biased by the changing contingencies, indicating that perceptual expectations are rapidly updated by associative learning and are used in perceptual inference.

To further corroborate the influence of associative learning on perception, we investigated an independent measure of learning, motor response speeds, and how this measure related to the observed changes in perception.We found that on unambiguous training trials, participants responded faster to sphere stimuli matching the currently predominant association than to stimuli not matching this association [613 vs. 642 ms ± 5 s.e.m, normalized difference −4.9 % ± 0.9 s.e.m., *t*_(30)_ = −5.40, *p* < 0.001], confirming that participants successfully tracked the changing associations and adapted their motor responses accordingly (den Ouden et al., 2010). Remarkably, across individuals the motor adaptation effect on unambiguous trials correlated with the perceptual bias on ambiguous trials (*r* = 0.42, *p* = 0.019, Pearson's product-moment correlation, Figure 2B). This result indicates a direct relationship between the effect of associative learning on motor response speeds and its influence on perception, suggesting that perceptual expectations induced by rapid associative learning indeed affect perceptual inference.

To verify that associative learning had an effect on perception independently from perceptual history, we conducted a trial-wise repeated measures logistic regression that included three regressors coding the expectations based on associative learning, priming, and sensory memory. This analysis revealed that all three regressors significantly predicted perception in the ambiguous test trials (“priming” *b* = 0.61 ± 0.10, Wald Chi-Square 36.4, *p* < 0.001; “sensory memory” *b* = 0.34 ± 0.07, Wald Chi-Square 26.2 *p* < 0.001, “associative learning” *b* = 0.11 ± 0.03, Wald Chi-Square 17.1, *p* < 0.001), indicating that associative learning impacted on the appearance of the ambiguous sphere stimuli despite the strong effects of perceptual history. Moreover, the full logistic regression with all three regressors compared to reduced logistic regressions with only two or one regressors was associated with lower QIC–value and hence better model fit (see Supplementary Table S1), further suggesting additive effects of associative learning, priming, and sensory memory.

### Experiment 2: expectation-induced perceptual biases

To further establish that perceptual expectations influenced perceptual inference from visual information rather than merely biasing participants' reporting behavior, we conducted Experiment 2 where we dissociated participants' perception from their key presses by the use of a variable stimulus-response mapping. We again found perception in ambiguous test trials to be biased toward the rotation direction of the preceding unambiguous training trial [“priming,” 39.6% ± 3.9 s.e.m., *t*_(30)_ = 10.10, *p* < 0.001, Figure 2C] and the preceding ambiguous test trial [“sensory memory,” 28.2% ± 4.6 s.e.m., *t*_(30)_ = 6.12, *p* < 0.001, Figure 2C]. Most critically and replicating our central finding of Experiment 1, in ambiguous test trials stimuli were perceived more frequently as rotating in the direction consistent with the currently predominant association than in the direction inconsistent with this association [6.2% ± 2.4 s.e.m., *t*_(30)_ = 2.59, *p* = 0.015, Figure 2C], and this effect was of intermediate size (Cohen's *d* = 0.5).

Again, we conducted a two-way ANOVA on the confidence rating data to test whether the found effect of associative learning on perception was due to a generally lower confidence in the perception of ambiguous stimuli (Supplementary Figure S2). This analysis revealed a main effect of stimulus type on confidence ratings, indicating that on ambiguous test trials compared to unambiguous training trials participants indicated less frequently the highest confidence level (*F* = 17.5, *p* < 0.001). There was also a main effect of associative learning (*F* = 5.1, *p* = 0.03), but no interaction between stimulus type and associative learning (*F* < 0.1, *p* = 0.98). The number of high-confidence rating trials was generally lower in Experiment 2 than in Experiment 1 (Supplementary Figure S2), which might be a result of the higher task demands associated with the variable stimulus-response mapping. However, participants still perceived the majority (more than 55%) of the ambiguous test trials at the highest confidence level (Supplementary Figure S1). Crucially, when including only test trials rated at the highest confidence level were included, learned associations still biased perceived rotation direction of the ambiguous stimuli (17.1% ± 6.2 s.e.m., *t*_(29)_ = 2.74, *p* = 0.011, one participant was excluded from this analysis because of not having rated any of the ambiguous test trials at the highest confidence level, Figure 2C).

Due to the higher task demands associated with the variable stimulus-response mapping we expected reaction times less likely to directly reflect associative learning. Nevertheless, we again observed an effect of associative learning on motor adaptation: in unambiguous trials participants were faster in making their perceptual decision about sphere stimuli matching the currently predominant association than to stimuli not matching this association (795 vs. 816 ms, normalized difference −2.5 % ± 1.1 s.e.m, *t*_(30)_ = −2.28, *p* = 0.030). Furthermore, there was a trend-wise correlation between the motor adaptation effect in unambiguous trials and the perceptual bias in ambiguous trials (*r* = 0.32, *p* = 0.075, Pearson's product-moment correlation, Figure 2D), corroborating the effect of rapidly changing perceptual expectations on perceptual inference.

As in Experiment 1, we used a repeated measures logistic regression to probe whether associative learning had an effect on perception independently from perceptual history. This analysis revealed that the effect of associative learning was present despite strong effects of perceptual history (“priming” *b* = 0.85 ± 0.11, Wald Chi-Square 60.0, *p* < 0.001; “sensory memory” *b* = 0.22 ± 0.04, Wald Chi-Square 27.8, *p* < 0.001, “associative learning” *b* = 0.10 ± 0.05,Wald Chi-Square 4.5, *p* < 0.034). Again, the full logistic regression with all three regressors compared to reduced logistic regressions with only two or one regressors was associated with a lower QIC–value and hence better model fit (see Supplementary Table S1). As key presses and perception were uncorrelated, these results indicate that perceptual expectations indeed influenced the perception of the ambiguous stimuli, rather than only biasing the participants' reporting behavior. This confirms that perceptual expectations used in perceptual inference dynamically change over time in response to both previous perceptual outcomes and associative context.

### Bayesian modeling of learning and inference

**Figure 3:**
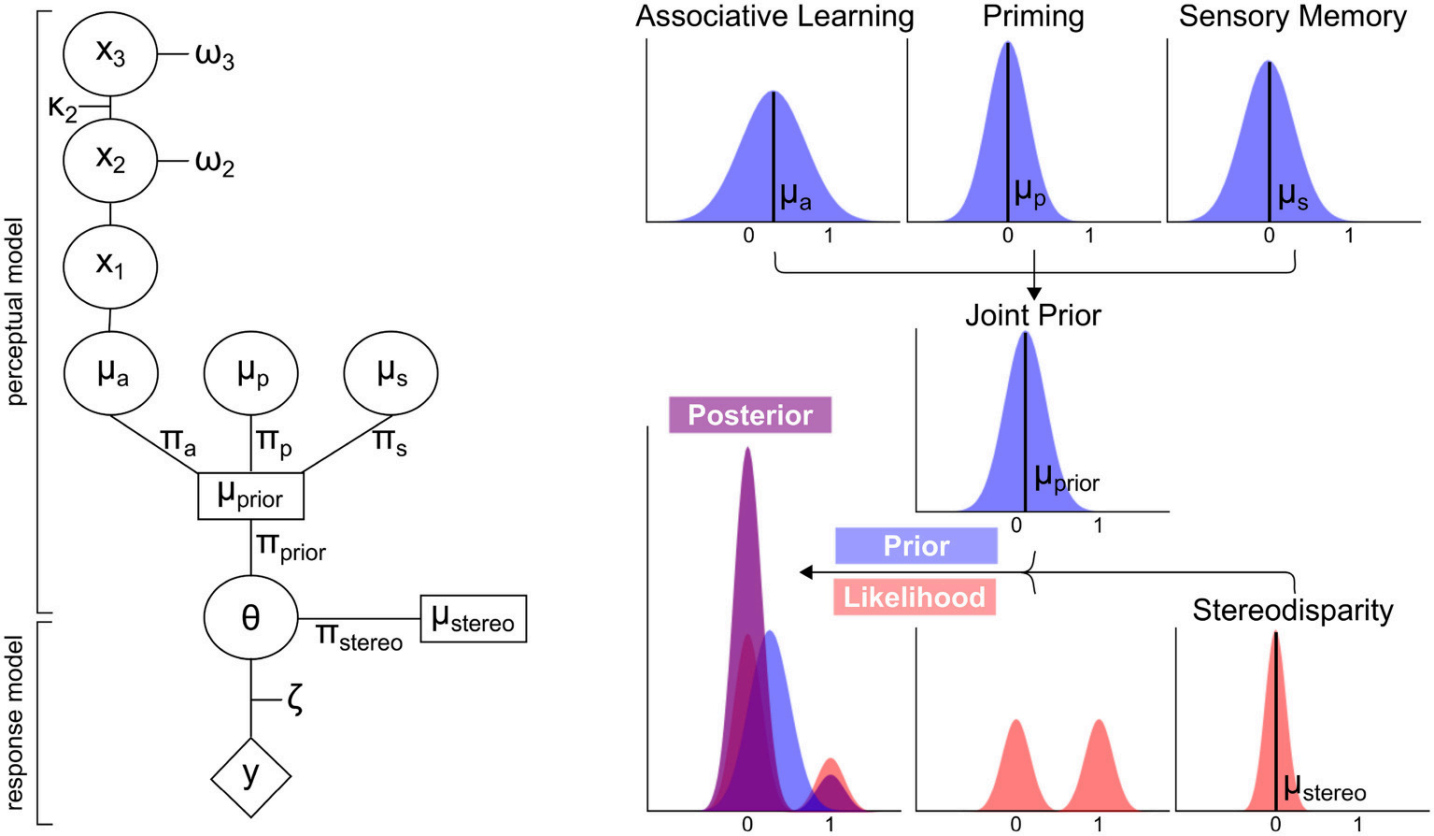
**Schematic overview of modeling analysis**. Left Panel: Perceptual and response model of modeling analysis. The perceptual model computes the means μ_*a*_, μ_*p*_ and μ_*s*_ and precisions π_*a*_, π_*p*_, π_*s*_ of the prior distributions “associative learning,” “priming,” and “sensory memory.” These are computed into a joint prior distribution with the mean μ_*prior*_ and π_*prior*_. Taking the stereodisparity information of the likelihood distribution into account (μ_*stereo*_ and π_*stereo*_), θ is derived to predict the responses *y*. π_*a*_, π_*p*_, π_*s*_ and π_*stereo*_ constitute the free parameters of the model. All other parameters are fixed to optimal values. Right Panel: Exemplary distributions for the prior distributions, stereodisparity information of the likelihood and resulting posterior distribution.

Our findings so far indicate that different aspects of prior experience such as perceptual history or associative context are flexibly used for the construction of visual perception, suggesting that perceptual expectations are continuously updated from different dynamic sources of information. In order to elucidate the underlying mechanisms, we applied a generic Bayesian model of learning and inference that rested on the trial-by-trial prediction of perceptual decisions (i.e., left- or rightward rotation) from posterior distributions (Figure 3). For every trial, these posterior distributions were derived by inference from likelihoods representing the ambiguous or unambiguous sensory information by weighted bimodal distributions (Sundareswara and Schrater, 2008) and prior distributions reflecting current perceptual expectations about perceptual outcomes (i.e., left- or rightward rotation) by unimodal distributions. Crucially, the influence of different dynamic aspects of prior experience on perceptual expectations was modeled by distinct prior distributions for “associative learning,” “priming,” or “sensory memory” that were separately updated on a trial-by-trial basis by learning (Mathys et al., 2011).

**Figure 4:**
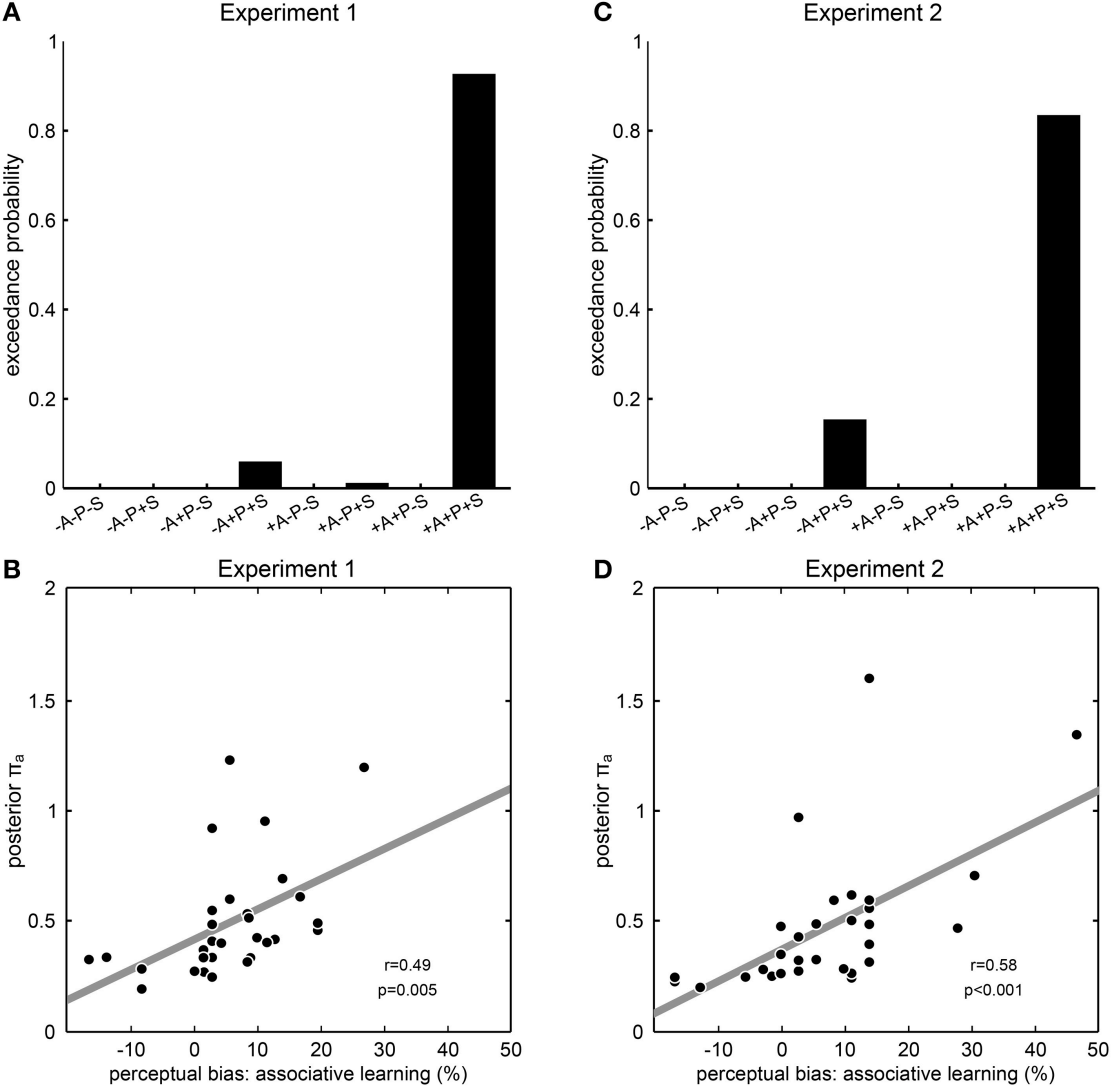
**Modeling results from Experiment 1 and 2**. **(A,C)** Results of model comparison. The model names at the y axis specify eight models with all possible combinations of the prior distributions “associative learning” (A), “priming” (P) and “sensory memory” (S), where “+” means that the corresponding prior distribution was included and “−” means that the corresponding prior distribution was excluded. Exceedance probabilities were derived by random-effects Bayesian model selection as implemented in SPM8. Exceedance probability refers to the probability that a model is more likely, at the group level, than any other model considered. In both experiments, the model +A+P+S including all prior distributions clearly outperformed all other models that lacked one, two or all of the prior distributions. **(B,D)** Correlation between model parameters and behavior. The posterior parameter estimates for the precision of the prior distribution “associative learning” from the winning model +A+P+S is correlated across participants with the perceptual bias induced by associative learning. Each dot represents one participant; the dashed line illustrates the fitted regression line; *r*- and *p*-values result from Pearson's product-moment correlations.

To investigate which dynamic aspects of prior experience would be incorporated into perceptual expectations and hence impact on perceptual inference, we constructed different models with any possible combination of the prior distributions “associative learning,” “priming,” or “sensory memory,” and compared them by random-effects Bayesian model selection. Consistent with our results so far, the model that included all three prior distributions was identified as a clear winning model in both Experiment 1 (exceedance probability 92.4%, Figure 4A) and Experiment 2 (exceedance probability 84.2%, Figure 4B), showing that a Bayesian model in which perceptual expectations were continuously updated from both associative context and perceptual history best explained participant's perceptual decisions. Qualitatively similar results were obtained when repeating the Bayesian modeling analysis with an alternative formulation of the prior distribution “associative learning” that took into consideration inter-individual differences in learning rate (Experiment 1: exceedance probability; Experiment 2: exceedance probability 89.6%; Supplementary Figure S3). To further examine the explanatory power of the winning model, we extracted the parameter encoding the precision of the prior distribution “associative learning” (π_*a*_) which we expected to reflect the impact of associative learning on perception. In line with this, we found a significant correlation across participants between this model parameter π_*a*_ with the participants' perceptual biases induced by associative learning from both Experiment 1 (*r* = 0.49, *p* = 0.005, Pearson's correlation, Figure 4C) and Experiment 2 (*r* = 0.58, *p* < 0.001, Figure 4D). Taken together, these results indicate a role of associative learning in addition to perceptual history in the continuous adaptation of perceptual expectations, suggesting that different aspects of prior experience are used to resolve perceptual ambiguity in a synergistic manner consistent with Bayesian learning and inference.

## Discussion

Our current results demonstrate that perceptual expectations strongly influence the perception of an ambiguous stimulus and that these expectations are shaped by fast associative learning as well as by perceptual history. Importantly, the influence of fast associative learning on perception had an intermediate effect size and was even present when participants were highly confident about their perception. Most critically, we show that perception of the ambiguous stimulus is best explained by a Bayesian model that continuously updates priors from both associative learning and perceptual history and combines them with the current sensory information in a probabilistic manner. Our current findings thus provide striking evidence that visual perception continuously incorporates different dynamic aspects of prior experience in a highly flexible manner consistent with Bayesian learning and inference, thereby offering a mechanistic explanation of how perceptual inference deals with the volatility of the environment.

The idea that previous experience influences on perception dates back to von Helmholtz (1867), and has lead to the notion perception does not reflect objective features of the environment but rather biological utility based on previous experience (Purves et al., 2015). In accordance with this, the mere experience of a stimulus improves or accelerates subsequent identification or detection of this stimulus (Schacter and Buckner, 1998), showing that perceptual history influences perceptual performance. Moreover, previous experience can also bias the appearance of both unambiguous stimuli (Fischer and Whitney, 2014) and ambiguous stimuli (Orbach et al., 1963; Maier et al., 2003), indicating an impact of perceptual history on the contents of perception. In this context, it is noteworthy that the appearance of ambiguous stimuli has been shown to be influenced by both prior experience evoked by the same ambiguous stimuli (“sensory memory”) as well as by prior experience of unambiguous versions of the ambiguous stimuli (“priming,” see Pearson and Brascamp, 2008 for a review). Our current results confirm the described effects of “priming” and “sensory memory” on the appearance of ambiguous stimuli. Most importantly, however, we formalized “priming” and “sensory memory” as continuously updated priors in the framework of Bayesian learning and inference, and found that a computational model that included both types of perceptual history best explained participants' perception. We therefore suggest that different types of perceptual history are at least in part separately built up and synergistically used to resolve perceptual ambiguity in a probabilistic fashion. This is in line with recent work showing independent effects of attentional priming and sensory memory on the perception of ambiguous stimuli (Brinkhuis et al., 2015).

It is well–established that in order to construct perception the visual system also relies on associative context. For example, when the shading of an object is consistent with two different shape interpretations, the visual system resolves this ambiguity by using the learned expectation that light usually comes from above (Sun and Perona, 1998). In this context, it is often implicitly assumed that perceptual expectations based on associative context are learned slowly across development until becoming hard-wired and fixed. Therefore, the question how such perceptual expectations adapt to the changing features of a dynamic environment has attracted interest only relatively recently. In this vein, recent studies have indicated that perceptual expectations can be modified through exposure to new statistical contingencies in the environment (Ernst et al., 2000; Adams et al., 2004; Flanagan et al., 2008; Chalk et al., 2010; Sotiropoulos et al., 2011), or placebo-like learning (Sterzer et al., 2008; Schmack et al., 2013), raising the idea that perceptual expectations based on associative context are less fixed than previously thought and remain plastic even in later life. The plasticity of perceptual expectations has been further corroborated by studies showing that repeated exposure to fixed cue-stimulus associations induces long-lasting perceptual changes in the perception of ambiguous stimuli (Sinha and Poggio, 1996; Haijiang et al., 2006; Di Luca et al., 2010). Here, we provide novel evidence demonstrating that perceptual expectations about appearance can be updated repeatedly and rapidly in accordance with changing cue-stimulus associations, which unequivocally speaks to an ongoing learning of perceptual expectations from associative context. Moreover, our computational modeling analyses revealed that a continuous update of prior probabilities based on associative learning well accounts for participants' perception, thereby providing a mechanistic description of the observed ongoing learning of perceptual expectations in terms of Bayesian learning and inference. Remarkably, treating associative learning, priming, and sensory memory as distinct, continuously updated priors which are integrated with sensory information constituted a plausible explanation of our participants' behavior. Our current results therefore illustrate how a generic Bayesian framework for learning and inference can explain the integration of different dynamic sources of information into perception.

We conclude that perceptual expectations that determine the contents of conscious perception flexibly capture various changing aspects of experience, indicating that the construction of our visual experience is a highly adaptive process ideally suited to deal with the volatile nature of the sensory environment in a probabilistic manner.

## Author contributions

KS participated in the design the study, supervised data acquisition, carried out the statistical analyses, interpreted the results, and drafted the manuscript. VW carried out the modeling analysis, interpreted the results, and drafted the manuscript. JH and KES participated in data analysis and interpretation, and critically revised the manuscript for intellectual content. PS conceived, designed and coordinated the study, and critically revised the manuscript for intellectual content. All authors gave final approval for publication.

## Funding

This project was funded by the German Research Foundation (grant STE-1430/7-1 to PS) and the German Federal Ministry of Education and Research within the framework of the e:Med research and funding concept (grant 01ZX1404A to KS). KS is participant in the Charité Clinical Scientist Program funded by the Charité - Universitätsmedizin Berlin and the Berlin Institute of Health. KES was supported by the René and Susanne Braginsky Foundation and the University of Zurich.

### Conflict of interest statement

The authors declare that the research was conducted in the absence of any commercial or financial relationships that could be construed as a potential conflict of interest.
