# Supplementary material for: Learning What to See in a Changing World
Source: Front Hum Neurosci. 2016 May 31;10:263. doi: 10.3389/fnhum.2016.00263 (PMC4885848; doi:10.3389/fnhum.2016.00263)
Supplement: Supplementary file 1 [file Image1.pdf]

SUPPLEMENTARY MATERIAL

Learning what to see in a changing world

Katharina Schmack, Veith Weilnhammer, Jakob Heinzle, Klaas Enno Stephan,

Philipp Sterzer

Inventory:

Supplementary Figures S1, S2 and S3

Supplementary Table 1

Supplementary Figures

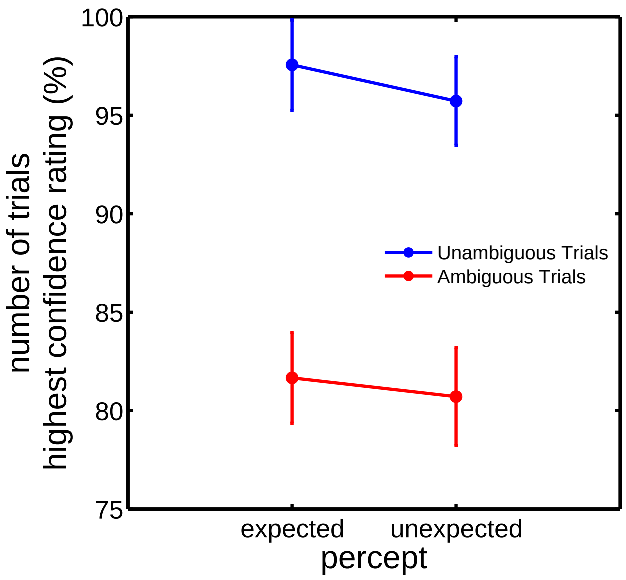

Supplementary Figure S 1: Effects of stimulus type and associative learning on the number of trials rated at the highest confidence level (Experiment 1). The red and blue data lines reflect the division of trials according to stimulus type into unambiguous and ambiguous trials. Along the x axis, trials are divided according to whether they were perceived in congruence with the currently predominant association (expected) or not (unexpected). On the y axis, the number of high-confidence trials is plotted. The number of high-confidence trials was calculated as the percentage of trials on which perception was rated as ’very sure’ with respect to all trials of the same condition (e.g. expected unambiguous trials). A two-way ANOVA showed a main effect of stimulus type (F=11.9, p=0.002). However, the majority (more than 75%) of ambiguous trials were still high-confidence trials, as it can be seen from the red line.

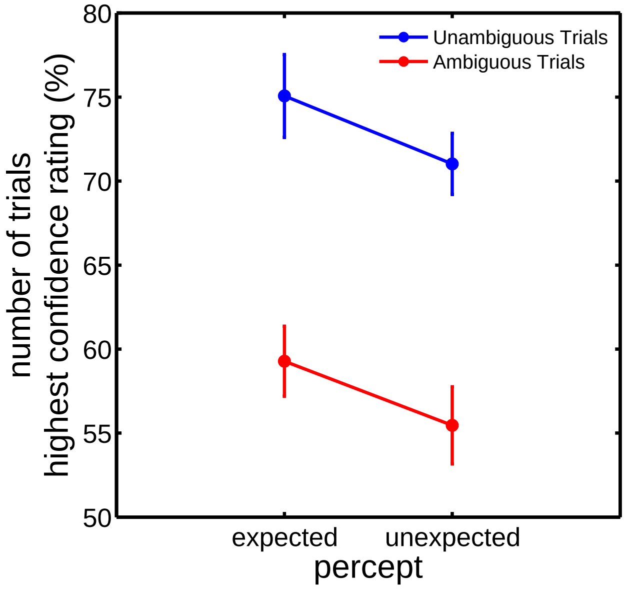

Supplementary Figure S 2: Effects of stimulus type and associative learning on the number of trials rated at the highest confidence level (Experiment 2). The red and blue data lines reflect the division of trials according to stimulus type into unambiguous and ambiguous trials. Along the x axis, trials are divided according to whether they were perceived in congruence with the currently predominant association (expected) or not (unexpected). On the y axis, the number of high-confidence trials is plotted. The number of high-confidence trials was calculated as the percentage of trials on which perception was rated as 'very sure' with respect to all trials of the same condition (e.g. expected unambiguous trials). Again, a two-way ANOVA showed main effect of stimulus type a main effect of stimulus type ($F=17.5$, $p<0.001$). Despite generally lower numbers of high-confidence trials in Experiment 2 than Experiment 1, the majority (more than 55%) of ambiguous trials were still rated at the highest confidence level, as it can be seen from the red line.

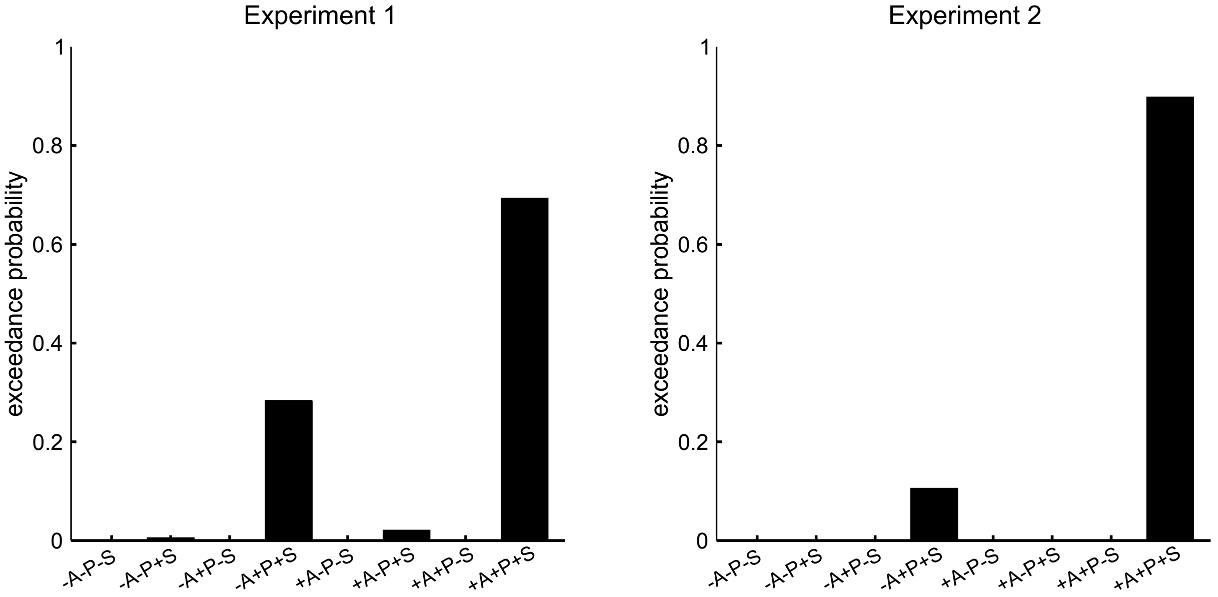

Supplementary Figure S 3: Results of Bayesian model comparison when accounting for inter-individual differences in learning rates from Experiment 1 and Experiment 2. Here, the prior distribution ’associative learning’ was derived by fitting a Bayesian learner with free time-invariant learning rate to the individuals’ behavior. The model names at the y axis specify eight models with all possible combinations of the prior distributions ’associative learning’ (A), ’priming’ (P) and ’sensory memory’ (S), where ’+’ means that the corresponding prior distribution was included and ’-’ means that the corresponding prior distribution was excluded. Exceedance probabilities were derived by random-effects Bayesian model selection as implemented in SPM8. Exceedance probability refers to the probability that a model is more likely, at the group level, than any other model considered. In both experiments, the model +A+P+S including all prior distributions won against all other models that lacked one, two or all of the prior distributions.

Supplementary Table

Supplementary Table 1: Model fit of different logistic regression models in Experiment 1 and Experiment 2. Given are quasi-likelihood under independence criterion (QIC) values, where lower QIC values indicate better model fit. Model name indicates the included regressors associative learning (A), priming (P), sensory memory (S). In both experiments, the full model ”A P S” is associated with the lowest QIC values and hence the best model fit.

Model name	Experiment 1	Experiment 2
**A P S**	**1345.5**	**1288.2**
A S	1418.2	1467.2
A P	1493.1	1346.4
S P	1348.7	1289.0
A	1580.9	1523.4
S	1421.4	1468.7
P	1495.6	1347.6
